# A Salinity Threshold Separating Fungal Communities in the Baltic Sea

**DOI:** 10.3389/fmicb.2019.00680

**Published:** 2019-03-29

**Authors:** Keilor Rojas-Jimenez, Angelika Rieck, Christian Wurzbacher, Klaus Jürgens, Matthias Labrenz, Hans-Peter Grossart

**Affiliations:** ^1^Department of Experimental Limnology, Leibniz-Institute of Freshwater Ecology and Inland Fisheries, Stechlin, Germany; ^2^Escuela de Biología, Universidad de Costa Rica, San José, Costa Rica; ^3^Chair of Urban Water Systems Engineering, Technical University of Munich, Munich, Germany; ^4^Leibniz Institute for Baltic Sea Research, Warnemünde, Germany; ^5^Institute for Biochemistry and Biology, University of Potsdam, Potsdam, Germany

**Keywords:** fungal diversity, baltic sea, salinity gradient, brackish waters, chytridiomycota, cryptomycota

## Abstract

Salinity is a significant factor for structuring microbial communities, but little is known for aquatic fungi, particularly in the pelagic zone of brackish ecosystems. In this study, we explored the diversity and composition of fungal communities following a progressive salinity decline (from 34 to 3 PSU) along three transects of ca. 2000 km in the Baltic Sea, the world’s largest estuary. Based on 18S rRNA gene sequence analysis, we detected clear changes in fungal community composition along the salinity gradient and found significant differences in composition of fungal communities established above and below a critical value of 8 PSU. At salinities below this threshold, fungal communities resembled those from freshwater environments, with a greater abundance of Chytridiomycota, particularly of the orders Rhizophydiales, Lobulomycetales, and Gromochytriales. At salinities above 8 PSU, communities were more similar to those from marine environments and, depending on the season, were dominated by a strain of the LKM11 group (Cryptomycota) or by members of Ascomycota and Basidiomycota. Our results highlight salinity as an important environmental driver also for pelagic fungi, and thus should be taken into account to better understand fungal diversity and ecological function in the aquatic realm.

## Introduction

In recent years, there has been a growing interest in studying fungi in the aquatic environment, mainly due to their significant roles in the cycling of nutrients and elements such as carbon (Wurzbacher et al., 2010; Grossart and Rojas-Jimenez, 2016). The increased use of next-generation sequencing approaches provides a detailed insight into biodiversity and occurrence of aquatic fungi (Wurzbacher and Grossart, 2012). Most studies coincide in pointing out a large number of fungal taxa to be described (Nikolcheva et al., 2003; Tedersoo et al., 2014; Wurzbacher et al., 2014; Richards et al., 2015; Grossart et al., 2016). Yet, the understanding of other aspects related to their metabolic functions and ecology remains to be investigated (Richards et al., 2015; Frenken et al., 2017).

Among factors modulating fungal diversity and community composition in aquatic ecosystems, salinity is of vital importance. For example, a clear differentiation between fungal populations in freshwater, estuarine, and marine environments has been observed (Booth and Kenkel, 1986; Geib et al., 2009; Mohamed and Martiny, 2011; Bálint et al., 2014; De Vargas et al., 2015; Jeffries et al., 2016; Zhang et al., 2016). Marine environments are generally characterized by a low proportion of fungi with respect to the overall number of eukaryotes (Richards et al., 2012, 2015). In relation to the composition of fungal communities in marine environments, some studies indicate that they are dominated by fungi belonging to the phylum Chytridiomycota, while other studies indicate that fungi belonging to Dikarya could be the most abundant (Hassett et al., 2017; Picard, 2017; Rämä et al., 2017; Wang et al., 2017). In freshwater ecosystems, there is usually a higher proportion of fungi belonging to Chytridiomycota (Comeau et al., 2016; Maier and Peterson, 2016; Wurzbacher et al., 2016; Arroyo et al., 2018).

Previous studies have shown that the majority of organisms are adapted to freshwater or marine conditions, while there are organisms like *Aspergillus flavus* that are ubiquitous and can inhabit both terrestrial and marine environments (Zuluaga-Montero et al., 2010; Ramírez-Camejo et al., 2012). Also, there are few brackish species with a wide tolerance to salinity (Logares et al., 2009; Telesh and Khlebovich, 2010). For example, Herlemann et al. (2011) showed that there are associations of brackish, marine and limnic bacteria in the Baltic, although their diversity was not affected and thus not significantly lower at brackish conditions. Despite this, the knowledge about fungal communities in brackish ecosystems and variations in their composition at different salinity levels is limited. First, because studies addressing these issues are rare (Hu et al., 2016; Kettner et al., 2017). Secondly, because most research has been conducted in estuaries with variable salinity conditions and short residence times, that do not select for populations adapted to constant brackish conditions along a more permanent and extended salinity gradient as can be found in the Baltic Sea.

**FIGURE 1:**
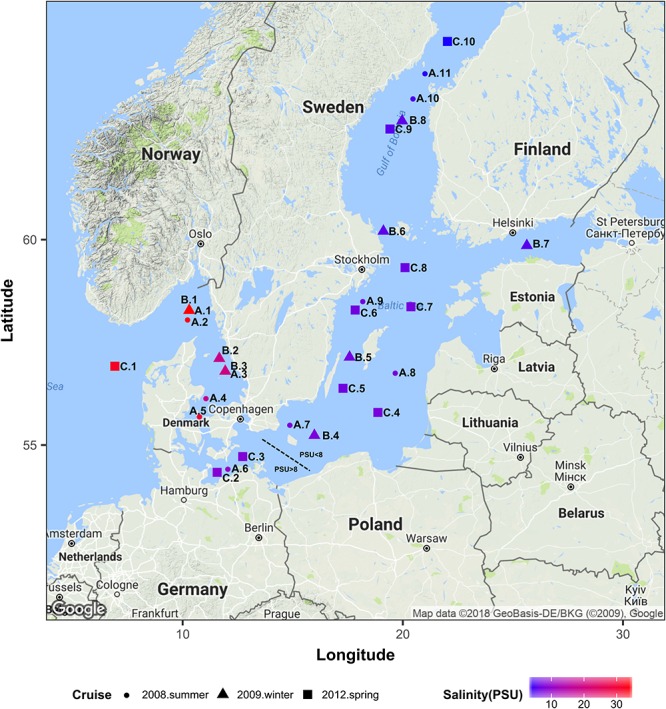
Geographical location of all sampling stations. Colors correspond to the surface water salinity. This map was generated using R ggmap package (Kahle and Wickham, 2013) importing images from Google maps. PSU, Practical Salinity Units.

The Baltic Sea constitutes an ideal model system to study autochthonous communities of brackish water fungi since it is one of the largest brackish ecosystems in the world with a progressive salinity decline over a distance of approx. 2000 km from 34 PSU in the Kattegat in the Southwest (Denmark–Sweden) to 3 PSU in the Bothnian Bay in the Northeast (Sweden–Finland; Figure 1). Since the narrow and shallow Danish straits constitute a barrier for the exchange with more saline waters from the open North Sea, the Baltic Sea has relatively stable horizontal and vertical salinity gradients, minimal tidal effects, and long retention times of up to 30 years (Kautsky and Kautsky, 2000; Reissmann et al., 2009). We provide new insights into the occurrence and diversity of aquatic fungi along the salinity gradient in the Baltic Sea and identify a threshold value at which fungal communities noticeably diverge.

## Materials and Methods

We used Illumina sequencing of the 18S rRNA gene to characterize the fungal community composition in the Baltic Sea, across three transects of ca. 2000 km each, and to determine the effect of salinity on structuring community composition along gradients ranging from 34 to 3 PSU (Figure 1). For this, we used two datasets of sequences. Dataset1 comprises 29 samples of surface waters collected during three cruises in summer 2008 (June 18th – July 14th), winter 2009 (February 23rd – March 9th) and spring 2012 (May 31st – June 8th). Dataset2 is composed of 36 samples collected at surface and intermediate layers (2–6 m vs. 11–65 m) from nine stations, during the spring 2012 cruise. At each sampling point of every cruise, water was collected using a conductivity /temperature /depth device (CTD, Sea-Bird Scientific, Bellevue, WA, United States) connected to polyethylene canisters. Concentrations of dissolved inorganic phosphate, nitrate, nitrite, silicate, ammonium and oxygen were analyzed as described by Grasshoff et al. (1983). Particulate organic carbon (POC) was determined as described by Rieck et al. (2015). Values of all measurements of each dataset are presented in Supplementary Figure S1.

For collection of nucleic acids, one liter of each water sample was filtered through 0.22 μm Durapore membranes (Merck, Germany) for all samples of dataset 1. In dataset 2, the same volume was filtered through 5.0 μm Nucleopore Track-Etched polycarbonate membranes (Whatman, Germany) and subsequently through 0.22 μm membranes to also distinguish between particle-associated (PA) and free-living (FL) eukaryotes. Filters were frozen in liquid nitrogen and stored at -80°C until further processing. DNA from the microorganisms on the filters was extracted using the phenol-chloroform protocol (Nercessian et al., 2005). With primers FF390 / FR1 (Prevost-Boure et al., 2011), we amplified the V7 and V8 regions of the 18S rRNA gene using 40 ng DNA as a template and the Fusion DNA Polymerase Herculase II (Agilent Technologies, United States). The PCR conditions consisted of 95°C for 3 min initial denaturation followed by 35 cycles at 95°C for 45 s, 53°C for 1 min, 72°C for 1 min, and a final extension at 72°C for 5 min. The ca. 360-bp-length amplicons were sequenced on a MiSeq sequencer with v3 2 × 300 nt chemistry (Illumina, San Diego, CA, United States). The sequence data are deposited in the NCBI Sequence Read Archive (**SRP126745**, BioSample accessions: **SAMN08707853–SAMN08707881**; **SAMN08707907–SAMN08707942**).

The 18S rRNA gene sequences were demultiplexed with Flexbar v3.0.3 (Dodt et al., 2012), paired and quality filtered (deltaq = 6) using Mothur v1.39.5 (Kozich et al., 2013). Reads shorter than 50 nucleotides and reads with more than 2% of ambiguities, or 2% of homopolymers, were excluded. Subsequent processing was performed with the SILVAngs v1.3 pipeline (Quast et al., 2013), including the alignment against the SILVA SSU rRNA SEED using SINA v1.2.10 (Pruesse et al., 2012), OTU clustering at a 0.03 distance cut-off with Cd-hit v3.1.2 (Li and Godzik, 2006), and taxonomic classification by local nucleotide BLAST search against SILVA SSU Ref dataset 132 using blastn (Camacho et al., 2009). The statistical analyses and visualizations were performed in R (R-Core-Team, 2015). We used Vegan (Oksanen et al., 2017) to calculate alpha diversity estimators, non-metric multidimensional scaling analyses, (NMDS), and the permutational analysis of variance (Permanova). To carry out the NDMS and Permanova, we used a table containing only the OTUs with abundances greater than 1 (singletons excluded), which was then transformed into a table of relative abundances. The NDMS was represented in a two-dimensional plot based on a Bray–Curtis similarity matrix. The statistical significance of the effects of the different variables on the fungal community composition was calculated using the adonis function with 999 permutations. *p*-Values of the pairwise Permanova were adjusted with the Benjamini–Hochberg method. The statistical differences in the diversity indices were estimated using the non-parametric Kruskal–Wallis test.

## Results and Discussion

In this work, we analyzed the diversity and composition of fungal communities along the Baltic Sea salinity gradient, using two different datasets of sequences of the 18S rRNA gene. The percentage of eukaryotic sequences belonging to the Fungal clade was 5.91% for dataset1 (surface water sampled in summer 2008, winter 2009, and spring 2012), and 7.77% for dataset2 (water sampled at different depths only in spring 2012). However, we observed marked variations in the relative proportion of fungi between sampling stations (Table 1 and Supplementary Figure S2). Within the 319 OTUs identified in dataset1, the two most abundant comprised 47.3% of the fungal sequences and were identified as LKM11 (Cryptomycota) and Rhizophydiales (Chytridiomycota). Within the 345 OTUs identified in dataset2, the two most abundant comprised 60.6% and were identified as *Paramicrosporidium* (Cryptomycota) and Rhizophydiales (Supplementary Table S1).

**Table 1:** Description of the datasets, number of samples, and proportion of fungal sequences relative to the total number of eukaryotic sequences.

Dataset	Description	Samples	Eukaryotes	Fungi	Percentage
Dataset1	Surface waters, 3 years-seasons: Summer 2008, Winter 2009, and Spring 2012	29	2,176,992	91,128	5.91 (range 0.23–30.5)
Dataset2	Two depths, particle-associated and free-living fractions, 1 year-season: Spring 2012	36	2,362,960	159,725	7.77 (range 0.26–68.1)


**FIGURE 2:**
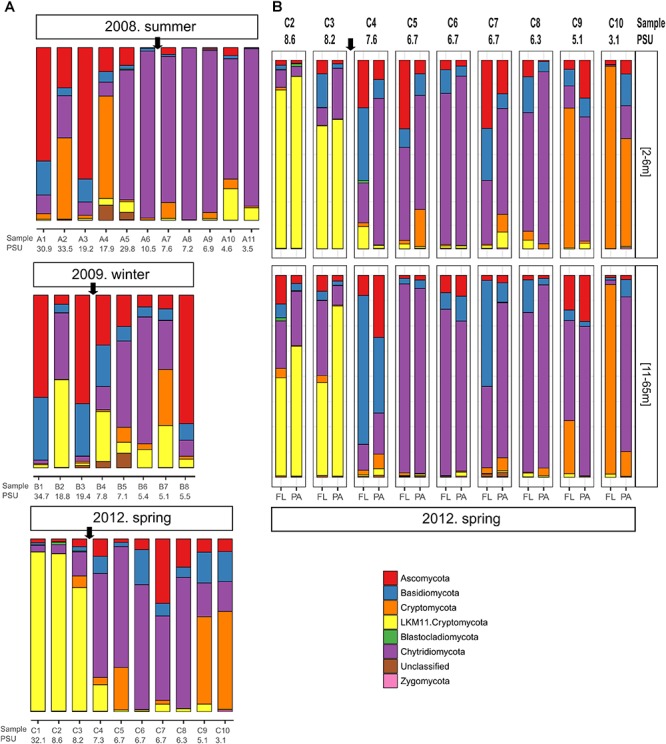
Fungal community composition along different transects and salinity gradients in the Baltic Sea. **(A)** Dataset 1 comprising 29 samples determined for surface waters collected during three cruises in summer 2008, winter 2009 and spring 2012. **(B)** Dataset 2 comprising 36 samples collected at nine stations during spring 2012 considering two depth horizons and free-living (FL) vs. particle-associated (PA) fractions. Sampling stations and salinities in PSU are shown. The arrow marks the limit between the stations above and below the 8 PSU.

**FIGURE 3:**
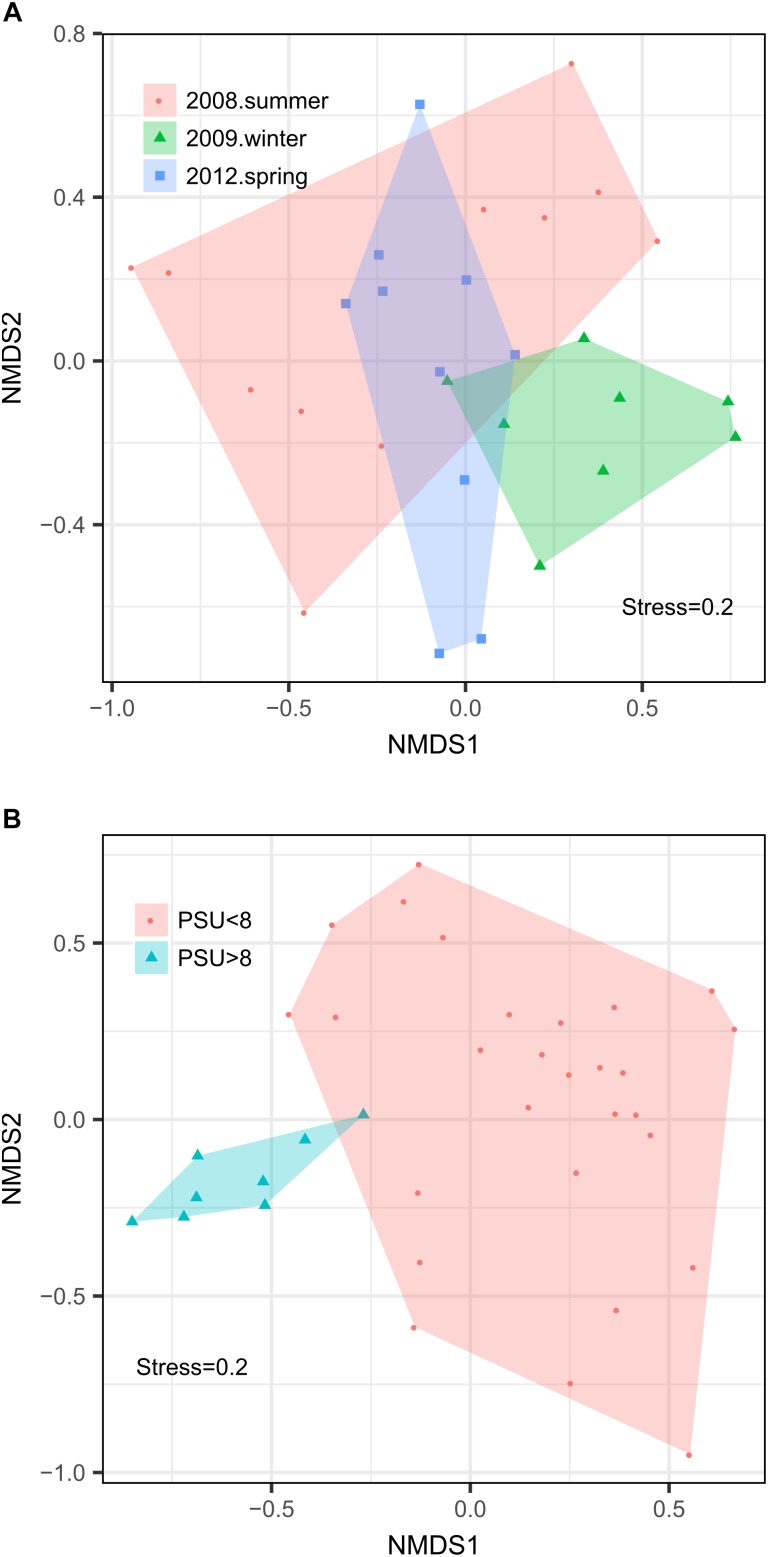
Non-metric multidimensional scaling analyses of the fungal communities in the Baltic Sea. The analysis was made from 65 samples of the two datasets used in this study: Dataset 1 comprising 29 samples determined for surface waters collected during three cruises in summer 2008, winter 2009, and spring 2012 and Dataset 2 comprising 36 samples collected at nine stations during spring 2012 considering two depth horizons and FL vs. PA fractions. **(A)** Analysis by season. **(B)** Analysis by salinity group.

We observed pronounced changes in the composition of the fungal communities along the Baltic salinity gradient (Figure 2), which was consistent at different phylogenetic levels. We also determined that the composition and abundance of OTUs in the communities above and below a threshold of 8 PSU were significantly different (Supplementary Table S2). For example, the divergence in fungal community composition at this salinity threshold was highly significant in dataset 2 (Permanova; *P* = 0.001, Figure 3B). This variation was consistent in the two depth layers analyzed of this dataset corresponding to the conditions of spring 2012. We also observed that a single strain of LKM11 was more abundant at sites with salinities above the threshold, as previously shown in other ecosystems and where presumably could have a saprophytic lifestyle (Rojas-Jimenez et al., 2017). In mesohaline waters, Chytrids assigned mainly to orders Rhyzophydiales and Lobulomycetales were more common, while a strain of *Paramicrosporidium* was more abundant at oligohaline conditions (Figure 2). In this dataset, we did not find significant differences between FL and PA fractions (Supplementary Table S2).

Changes in fungal community composition above vs. below the salinity threshold were also significant in dataset 1 (Permanova, *P* = 0.008). In this dataset, we further determined significant differences in community composition between the three studied seasons (Permanova, *P* = 0.026, Figure 3A). In summer 2008, the sites with higher salinities showed a greater abundance of Ascomycota, particularly of the genera *Cladosporium* and *Saccharomyces*, while the mesohaline sites were dominated by Chytridiomycota belonging to the orders Rhizophydiales and Gromochytriales. In winter 2009, the pattern was more variable, although a greater abundance of Leotiomycetes (Ascomycota) and Cystobasidiomycetes (Basidiomycota) was observed at salinities above 8 PSU. Below this threshold, taxa belonging to the chytrid orders Rhizophydiales and Lobulomycetales were more abundant, except at station B.8 (Bothnian Gulf) where *Geotrichum* (Ascomycota) was more frequent (Figure 2).

Although not yet shown for fungal communities, the notion of a specific salinity threshold driving variations in organismic community composition is not entirely new. For example, Telesh and Khlebovich (2010) proposed a critical range of 5–8 PSU for zooplankton communities in the Baltic Sea, while Herlemann et al. (2011) also observed that bacterial communities formed different clusters above and below a salinity of 7.7 PSU. In addition, previous studies on aquatic fungi have shown differences along very broad salinity gradients, e.g., between estuarine, coastal, and oceanic samples (Jeffries et al., 2016); stream, pond, melting ice, and estuary samples in the Arctic (Zhang et al., 2016); between the freshwater mixolimnion and brackish monimolimnion in Antarctic lakes (Rojas-Jimenez et al., 2017); salty, brackish, and freshwater marshes (Mohamed and Martiny, 2011); intertidal wetlands and shallow marine sediments (Picard, 2017); and a coastal environment with variable riverine inputs (Taylor and Cunliffe, 2016).

Despite the overall changes in the eukaryotic community composition between seasons, we show that a specific salinity threshold is related to variations in the composition of pelagic fungal communities, particularly in a system with a high residence time such as the Baltic Sea. This is consistent with earlier studies, e.g., Shearer et al. (2007) showed that taxonomic occurrence and species distribution were mainly determined by temperature and salinity, while Tisthammer et al. (2016) showed that global biogeography of marine fungi is shaped by local environmental variables, including salinity. We found some significant effects of other environmental variables, such as SiO_2_, total N, O_2_, and PO_4_ on the structure of the communities, however, these effects occurred in a rather inconsistent way, being present in one or the other data sets analyzed (Supplementary Table S3). Thus, for a better mechanistic understanding we consider that it is important to investigating the effects of salinity and other environmental variables on the structure of aquatic fungal communities in a higher spatio-temporal resolution. This is of particular importance in transition zones such as between the North Sea and the Baltic Sea, where changes in these variables are quite abrupt.

The specific causes of the observed greater or lower tolerance to salinity of specific fungal phylotypes remain to be elucidated. However, there are three possible physiological mechanisms that can intervene: The first is related to the increase in the concentration of ions within the cells through the use of high affinity transport systems that allow to carrying, i.e., potassium ions inward and then confine them in vacuoles. This mechanism allows tolerant fungi to maintain an osmotic potential lower than the external water potential. The second mechanism is related to the osmoregulatory capacity, which is, to adjust the accumulation of specific solutes, called osmolytes or osmoprotectants, through endogenous production or by uptake from the medium. The glycerol is one of the main osmoprotectant in fungi, is harmless for the structure and function of cytoplasmic proteins and also alleviates some inhibitory effects of high ionic strength. The two previous mechanisms are present in both yeasts and filamentous fungi (Jennings, 1983; Blomberg and Adler, 1992; Logares et al., 2009). The third mechanism is related to a mechanical strengthening of the cell and is present mainly in filamentous fungi, where for example, increases in the thickness of cell walls have been observed with increases in salinity (Ahumada-Rudolph et al., 2019). On the other hand, it is well known that chytrids prefer aquatic environments with low osmotic potentials, probably because their zoospores lack a cell wall, which make them more susceptible to osmotic stress (Gleason et al., 2008; Gleason and Lilje, 2009). However, some chytrids have been reported as parasites of small green algae and diatoms in marine waters, suggesting that even these organisms may be using other mechanisms to cope with salinity (Gleason et al., 2011; Hassett et al., 2017).

It is also possible that the range of distribution of some fungal groups, particularly those that contain phytoplankton parasites such as Rhizophydiales, Lobulomycetales, and Gromochytriales (Frenken et al., 2017) are affected by the distribution of their respective hosts. This would be consistent with studies showing the Darss Sill (with salinities between 8 and 10 PSU) as the border for some phytoplankton species (Lemke et al., 1994; Witkowski et al., 2005; Wasmund et al., 2011; Celepli et al., 2017). Conversely, members of Basidiomycota and Ascomycota, such as unicellular yeasts, can be more tolerant to higher levels of salinity, as observed during summer and winter at stations with salinities > 8 PSU (Kutty and Philip, 2008).

In this study, we estimated that the average richness per sample comprises 46 fungal OTUs (range 14–97) in dataset 1 and 47 OTUs (range 20–76) in dataset 2 (Supplementary Figure S1). Within dataset 1, we did not find any significant differences in richness values, season of the year, nor between communities at salinities above and below 8 PSU (Kruskal–Wallis, *P* > 0.05). We also did not observe significant differences in fungal richness of dataset2 when considering variables such as salinity, depth, or between the PA and FL fungal fractions. Similar patterns of the Shannon index were visible with mean values of 1.71 and 1.59, for datasets 1 and 2, respectively. Again, no significant differences were found with any of the environmental variables analyzed. From these results we conclude that salinity has a clear effect on fungal community composition in the Baltic Sea, which is not necessarily reflected in changes of the species richness and diversity.

Based on the obtained taxonomic identification and related ecological information, we were able to recognize members of Ascomycota, Basidiomycota, and Zygomycota as saprotrophs. We speculate that these fungi participate in the recycling of nutrients and biodegradation of recalcitrant compounds, or can act as biotrophs of cyanobacteria, algae, oomycetes, protozoa, and microinvertebrates (Kutty and Philip, 2008; Leshem et al., 2016). Other members of Chytridiomycota, particularly those assigned to Rhyzophydiales and Lobulomycetales, might act as parasites (Frenken et al., 2017). They can infect unpalatable silicon-covered diatoms, and release energy-rich zoospores, that can be readily utilized by fungal predators (Kagami et al., 2007, 2011, 2014). Additionally, some of these parasitic fungi have the potential to inflict mass mortalities on hosts, because changes in phytoplankton size, and suppress bloom events, like those occurring in spring or late summer (see review Frenken et al., 2017). However, we request to further examine the functional and ecological roles of fungi in the Baltic Sea, using cultivation-based methods to uncover their different metabolic potential.

## Conclusion

Our work confirms that salinity holds an important role in structuring pelagic fungal communities of the Baltic Sea. In particular, we determined a significant variation in fungal species composition and community structure above and below a threshold of 8 PSU. This finding has important implications since the presence of different fungal groups with changing salinity levels potentially influence aquatic food web structure and ecosystem functions. In the future, it will be necessary to analyze a greater number of sampling points along the gradient, also considering a higher temporal resolution, which would allow a better understanding of the fluctuations of fungal populations in time and space. Likewise, we consider it important to study in greater detail the main physiological mechanisms of adaptation to salinity that operates in the different phylogenetic groups of fungi.

## Originality-Significance Statement

In three expeditions to the Baltic Sea, carried out during different years and seasons, we explored the diversity and composition of fungal communities along transects of ca. 2000 km and a salinity gradient ranging from 34 to 3 PSU. Sequence analysis of the 18S rRNA gene shows that salinity is important for structuring the pelagic fungal communities in the Baltic. We determined a significant variation in the composition of fungal species above and below a threshold of 8 PSU. We highlight salinity as an important environmental factor that should be taken into account to better understand the fungal diversity and their ecological functions in the aquatic, pelagic environment.

## Data Availability

The datasets generated for this study can be found in NCBI Sequence Read Archive, SAMN08707853–SAMN08707881 and SAMN08707907–SAMN08707942.

## Author Contributions

KR-J, ML, CW, and H-PG designed the study. CW and AR collected the sample. CW, KJ, and KR-J performed the analysis. KR-J and H-PG wrote the manuscript. All authors helped to revise the manuscript.

## Conflict of Interest Statement

The authors declare that the research was conducted in the absence of any commercial or financial relationships that could be construed as a potential conflict of interest.
