# Supplementary material for: A Salinity Threshold Separating Fungal Communities in the Baltic Sea
Source: Front Microbiol. 2019 Mar 29;10:680. doi: 10.3389/fmicb.2019.00680 (PMC6449873; doi:10.3389/fmicb.2019.00680)

Supplementary Information

A salinity threshold separating fungal communities in the Baltic Sea

Keilor Rojas-Jimenez[1,2], Angelika Rieck[1], Christian Wurzbacher[1,3], Klaus Jürgens[4], Matthias Labrenz[4], Hans-Peter Grossart[1,5]

[1]Department of Experimental Limnology, Leibniz-Institute of Freshwater Ecology and Inland Fisheries, Alte Fischerhuette 2, D-16775 Stechlin, Germany

[2]Escuela de Biología, Universidad de Costa Rica, 11501 San José, Costa Rica

[3]Chair of Urban Water Systems Engineering, Technical University of Munich, Am Coulombwall 3, 85748 Garching, Germany

[4]Leibniz Institute for Baltic Sea Research, Warnemünde, Germany

[5]Institute for Biochemistry and Biology, Potsdam University, Maulbeerallee 2, 14469 Potsdam, Germany

Corresponding author

Hans-Peter Grossart

Alte Fischerhuette 2, D-16775 Stechlin, Germany

Email: hgrossart@igb-berlin.de

Tel.: +49 (0)33082 699 91

Fax: +49 (0)33082 699 17

Table S1. Taxonomy and relative abundance of the 10 most common OTUs of each dataset.

OTU-Nr.	Rel. Abund.	Taxonomy
**Dataset1**		
otu038	24.41	Cryptomycota / LKM11
otu028	22.87	Chytridiomycota / Rhizophydiales
otu032	7.81	Chytridiomycota / Chytridiomycetes
otu013	6.59	Chytridiomycota / Gromochytriaceae
otu036	5.39	Cryptomycota / Paramicrosporidium
otu025	4.43	Chytridiomycota / Rhizophydiaceae
otu121	3.26	Ascomycota / Helotiales
otu024	2.55	Chytridiomycota / Kappamycetaceae
otu019	2.46	Chytridiomycota / Lobulomycetaceae
otu269	2.30	Basidiomycota / Cystobasidiomycetes
**Dataset2**		
otu031	42.10	Cryptomycota / Paramicrosporidium
otu024	18.52	Chytridiomycota / Rhizophydiales
otu033	13,62	Cryptomycota / LKM11
otu015	6.60	Chytridiomycota / Lobulomycetaceae
otu028	2.56	Chytridiomycota / Chytridiomycetes
otu022	2.11	Chytridiomycota / Rhizophydium
otu200	2.04	Ascomycota / Saccharomyces
otu308	1.84	Basidiomycota / Rhodotorula
otu310	1.07	Basidiomycota / Coleosporium
otu135	0.71	Ascomycota / Helotiales

Table S2. Statistical analysis (PERMANOVA) of the fungal community composition related to differences in the measured environmental variables. Asterisks indicate significant differences.

Dataset	Variable	F.Model	R2	p.value
**Dataset1 (interanual 2008, 2009, 2012)**	Salinity* (<8 vs >8 PSU)	2.304	0.079	0.008
	Season*	1.767	0.120	0.026
**Dataset2 (spring 2012)**	Salinity* (<8 vs >8 PSU)	13.302	0.281	0.001
	Fraction (FL vs PA)	2.034	0.056	0.072
	Depth (2-6 vs 11-65m)	0.604	0.017	0.742

Table S3. Statistical analysis (PERMANOVA) of the fungal community composition related to additional environmental variables. The cutoff values are the median of each variable within each data set.

Dataset	Variable	F.Model	R2	p.value
**Dataset1 (interanual 2008, 2009, 2012)**	SiO_2 (>/< 10.3 µM)	1.1761	0.04174	0.2687
	Total N (>/< 0.37 µM)	2.2946	0.07833	0.01598
	O_2 (>/< 8.02 ml/L)	1.5413	0.054	0.1239
	PO_4 (>/< 0.22 µM)	1.4934	0.05241	0.1399
**Dataset2 (spring 2012)**	SiO_2 (>/< 11.8 µM)	2.4834	0.06807	0.03097
	Temperature (>/< 7.6 °C)	4.6407	0.1201	0.003996
	Total N (>/< 0.28 µM)	2.4834	0.06807	0.02597
	O_2 (>/< 8.12 ml/L)	6.55	0.16153	0.000999
	PO_4 (>/< 0.36 µM)	2.9819	0.08063	0.00999
	Chla (>/< 1.28 mg/m3)	1.0973	0.03126	0.3497

Figure S1. Environmental parameters determined at stations along three transects of ca. 2000 km in the Baltic Sea. **A)** Dataset 1: values of 29 samples determined for surface waters collected during three cruise expeditions in summer 2008, winter 2009, and spring 2012. **(B)**. Dataset 2: points correspond to mean values while the bars represent minimum and maximum values. This dataset is composed of 36 samples collected at nine stations during spring 2012 considering two depths and two fractions (particle-associated and free-living). Total N represents the sum of NO_2, NO_3, and NH_4.

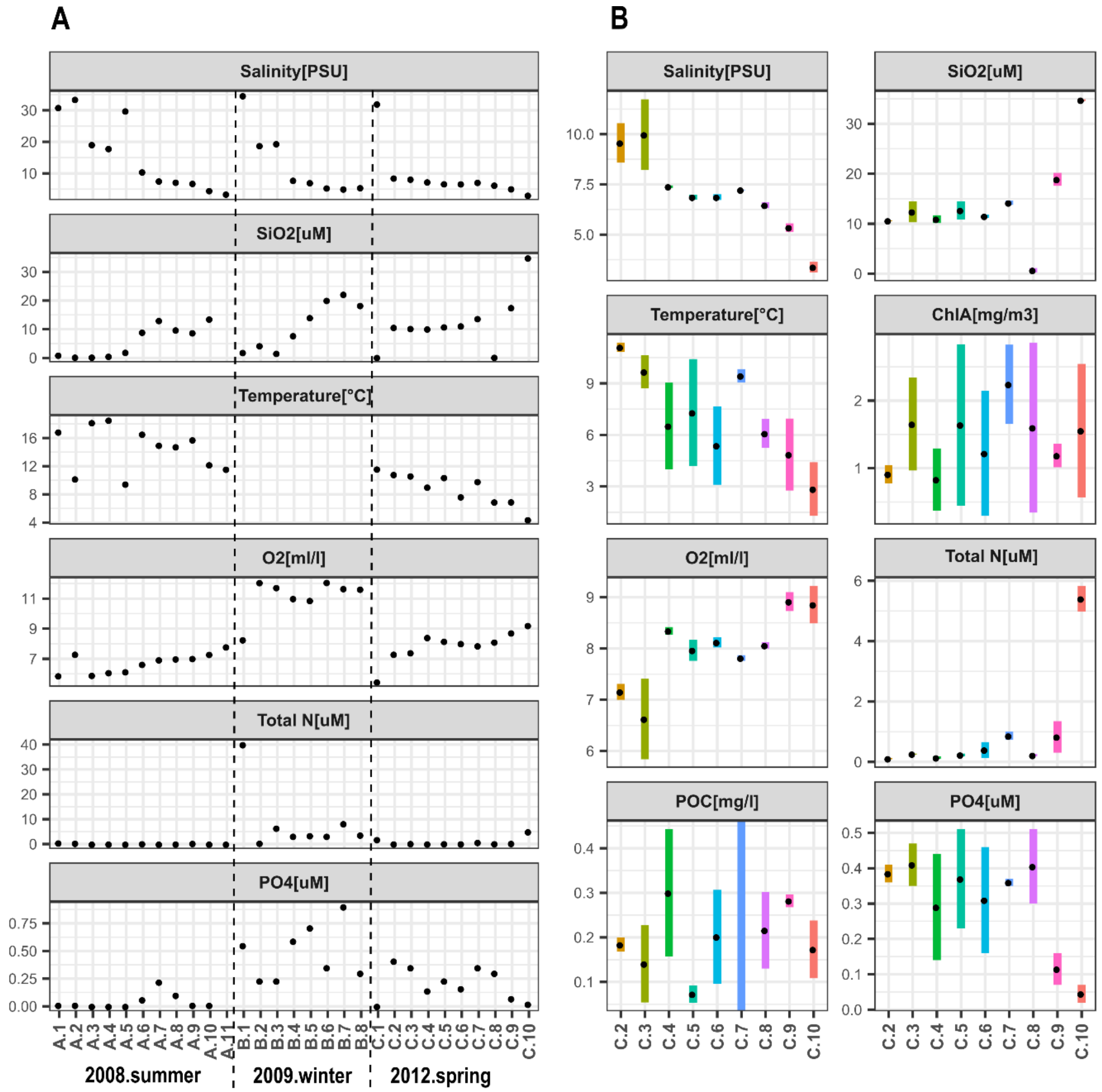

Figure S2. Proportion of sequences assigned to the Fungal Kingdom respect to the total number of eukaryotic sequences, estimation of the richness of OTUs per sample, and alpha diversity according to the Shannon Index. Panel A shows the estimations of the inter-annual dataset1 while panel B shows the estimations of the dataset2 sampled in spring 2012. Error bars represent the standard deviation.

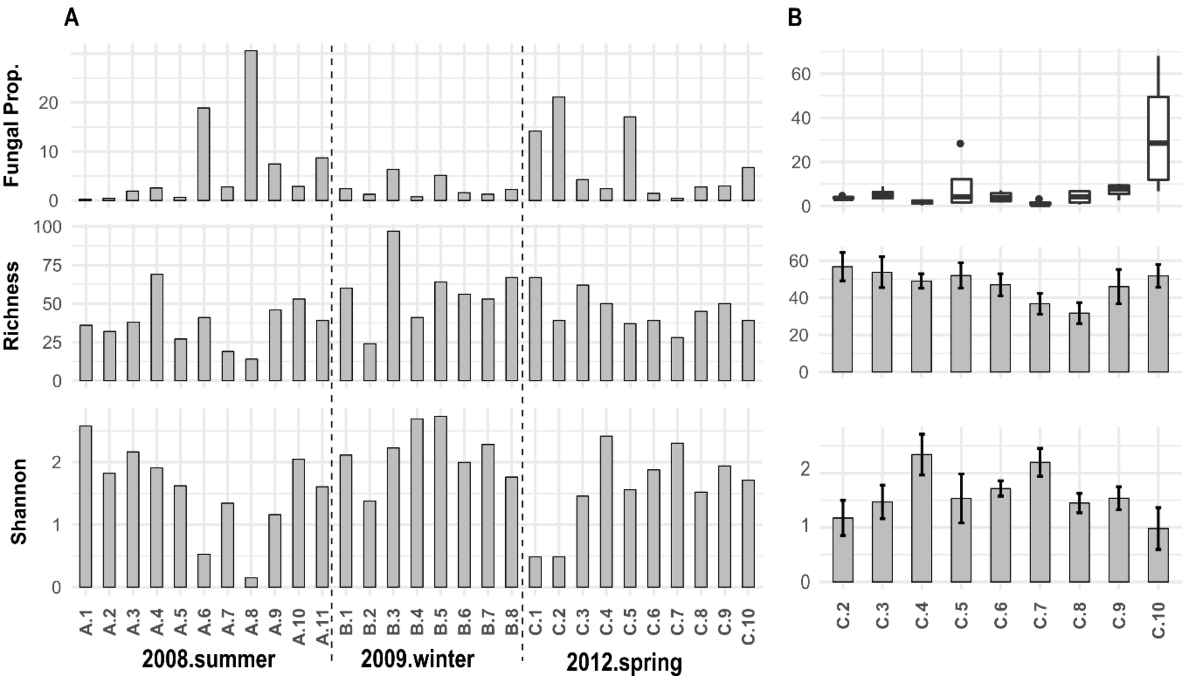

Supplement: Supplementary file 1 [file Data_Sheet_1.PDF]
